# Osteoporosis in Patients with Pre-Existing Diabetes Mellitus and in Women with Estrogen Deficiency: A Molecular and Cellular Perspective

**DOI:** 10.3390/ijms27031453

**Published:** 2026-01-31

**Authors:** Chin-Yen Pang, Li-Ru Chen, Kuo-Hu Chen

**Affiliations:** 1 Department of Physical Medicine and Rehabilitation, Mackay Memorial Hospital, Taipei 10421, Taiwan; ian61015@gmail.com (C.-Y.P.); gracealex168@gmail.com (L.-R.C.); 2 Department of Mechanical Engineering, National Yang Ming Chiao Tung University, Hsinchu 30009, Taiwan; 3 Department of Obstetrics and Gynecology, Taipei Tzu-Chi Hospital, The Buddhist Tzu-Chi Medical Foundation, New Taipei City 23142, Taiwan; 4 School of Medicine, Tzu-Chi University, Hualien 97004, Taiwan

**Keywords:** osteoporosis, diabetes mellitus-related osteoporosis, estrogen deficiency-induced osteoporosis, treatment of osteoporosis

## Abstract

Osteoporosis is a prevalent metabolic bone disorder characterized by reduced bone mass, compromised microarchitecture, and increased fracture risk. Its pathogenesis extends beyond simple bone mineral density (BMD) loss and reflects complex disruptions in bone remodeling governed by osteoblast–osteoclast coupling and systemic metabolic factors. This review lays particular emphasis on diabetes mellitus-related osteoporosis (DOP) and estrogen deficiency-induced osteoporosis (EDOP), discussing bone remodeling between osteoclastogenesis and osteoblast differentiation regulated by key signaling pathways, including the RANKL/RANK/OPG, Wnt/β-catenin, BMP–Smad, Hedgehog, and inflammatory cytokine networks. This review then explores how chronic hyperglycemia, insulin deficiency or resistance, oxidative stress, ferroptosis, advanced glycation end products, and low-grade inflammation disrupt bone homeostasis in diabetes, resulting in impaired bone quality and elevated fracture risk, particularly in type 2 diabetes. In parallel, we discuss the genomic and non-genomic actions of estrogen in maintaining skeletal integrity and elucidate how estrogen deficiency accelerates bone resorption and suppresses bone formation through altered cytokine signaling, oxidative stress, and impaired mechanotransduction. Advances in diagnostic strategies beyond BMD, including trabecular bone score, high-resolution peripheral quantitative computed tomography, and emerging biomarkers, are reviewed. Finally, this review summarizes current and emerging therapeutic approaches tailored to DOP and EDOP, emphasizing the need for mechanism-based, individualized management. A deeper understanding of these shared and distinct pathways may facilitate improved risk stratification and the development of targeted interventions for osteoporosis.

## 1. Introduction

Osteoporosis is a chronic metabolic bone disorder characterized by reduced bone mass and microstructural deterioration of bone tissue, leading to increased bone fragility and a heightened risk of fractures [1]. Globally, approximately half of women aged 50 and older, and one-fifth of men, are at risk of developing osteoporosis or experiencing fragility fractures [2].

Osteoporosis is classified into two types based on its etiology: primary and secondary. Primary osteoporosis results from aging or menopause-associated bone loss and can be further divided into two subtypes. Type I, or postmenopausal osteoporosis, is caused by estrogen deficiency, while Type II, or senile osteoporosis, typically occurs in individuals over the age of 75 and affects both sexes due to aging. In contrast, secondary osteoporosis arises from causes other than aging or menopause, such as medication use or underlying pathological conditions [3].

Bone strength—and thus fracture risk—depends on various bone properties, with bone mineral density (BMD) being the most commonly measured parameter. Expressed as grams of mineral per area or volume, BMD peaks around the age of 30 and gradually declines thereafter [4]. Although BMD is a valuable and easily measured marker for osteoporosis evaluation, studies have shown that treatment decisions should not rely solely on BMD, but should also consider other risk factors to more effectively prevent osteoporotic fractures [5,6].

Numerous risk factors contribute to the development of osteoporosis. Non-modifiable risk factors include age, ethnicity, a family history of osteoporosis, postmenopausal status, and the presence of rheumatoid arthritis in men. Modifiable risk factors include low body weight, sedentary lifestyle, diabetes mellitus, vitamin D insufficiency, chronic steroid use, smoking, and alcohol consumption [3,4,7,8,9,10].

In this review article, we introduce the mechanisms of bone remodeling and explore how diabetes mellitus and estrogen deficiency contribute to osteoporosis from both molecular and cellular perspectives.

## 2. Methods of Literature Review

The literature was searched to identify basic and clinical research that investigated the molecular and cellular mechanisms as well as clinical treatments of osteoporosis in patients with pre-existing diabetes mellitus and women with estrogen deficiency. For the current review examining the etiology of osteoporosis as well as its treatment, all relevant articles were collected from the databases Ovid Medline and PubMed using the search terms “osteoporosis”, “diabetes mellitus related osteoporosis”, “estrogen deficiency induced osteoporosis”, and “treatment of osteoporosis”. For screening and inclusion in the second stage, only English-language articles with available full text were considered for inclusion in further analyses. Duplicate articles were also excluded in this stage.

In the next stage, two experts in the field individually reviewed all retrieved articles to exclude studies with poor research design, questionable methodology, or unclear outcomes, thereby ensuring the quality of retrieved research. Finally, a total of 83 articles were deemed eligible for inclusion in this review.

## 3. Cellular and Molecular Mechanisms of Osteoporosis: Bone Modeling

### 3.1. Structural and Cellular Components of Bone

Bone is a dynamic connective tissue composed of organic and inorganic components. The organic matrix consisting primarily of type I collagen, which accounts for about 90% of the matrix, together with various non-collagenous proteins, supports bone formation and remodeling [11]. The inorganic portion, primarily hydroxyapatite crystals composed of calcium and phosphate, provides rigidity and accounts for most of the bone’s mass [12].

There are two types of bone, cortical bone and trabecular bone, distinguished by their organization. Cortical (compact) bone, which presents with a dense and ordered structure, accounts for approximately 80% of bone mass. The rest is trabecular (spongy) bone, which is porous and metabolically active. Structurally, bone consists of osteons—units containing Haversian canals, lamellae, and osteocytes interconnected by canaliculi [12,13].

Bone structure is regulated by four main cell types. Osteoblasts build bone, osteoclasts resorb it, osteocytes maintain bone homeostasis by sensing mechanical stress, and bone lining cells cover the bone surface during the quiescent phase.

Together, these components support bone’s structural integrity, metabolic activity, and continuous remodeling [13,14].

### 3.2. Bone Homeostasis

Since micro-fractures often result from daily stress on the bone, the bone remodeling process is critically important. Bone remodeling follows a specific sequence and lasts about 6 months. There are four stages which include (1) activation of osteoclast precursors into multinucleated osteoclasts, (2) resorption of bone by osteoclasts, (3) reversal of the resorption signal and recruitment of osteoblasts, and (4) the formation of new bone by osteoblasts [14,15,16].

For the remainder and the majority of the bone structure, both osteoblasts and osteoclasts remain in the quiescence stage, which is inactive in bone formation and resorption [15].

### 3.3. The Regulation of Bone Remodeling in Healthy and Osteoporotic Populations

Osteoporosis results from an imbalance of normal bone remodeling, in which bone resorption is favored over bone formation. The bone remodeling cycle is regulated by many factors including hormone signals, paracrine and autocrine factors, and the mechanical loading of bone. Additionally, a range of systems—endocrine, immune, nervous, and others—are involved in the regulation of bone remodeling [17]. To further understand the mechanisms linking menopause and diabetes mellitus to osteoporosis, a more detailed understanding of the underlying signaling pathway is needed. Herein, we focus on the signaling pathways that primarily control osteoclast and osteoblast activity.

### 3.4. Osteoclast Differentiation and Regulation of Bone Resorption

**Figure 1:**
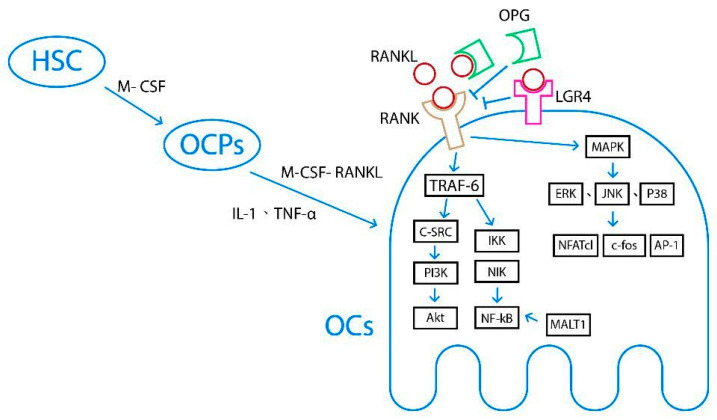
The underlying mechanisms of osteoclast differentiation and the regulation of bone resorption, including the RANKL/RANK/OPG signaling pathway and the IL-1/TNF-α signaling pathway. HSC: hematopoietic stem cell; OCPs: osteoclast precursor cells; OCs: osteoclasts; RANK: receptor activator of NF kappa B; RANKL: receptor activator of NF kappa B ligand; OPG: osteoprotegerin; LGR4: leucine-rich G-protein-coupled receptor 4; TRAF-6: tumor necrosis factor receptor-related factor-6; MAPK: mitogen-activated protein kinase.

Osteoclasts are the primary functional cells involved in bone resorption. They are derived from osteoclast precursor cells (OCPs) which originate from the monocyte/macrophage lineage of hematopoietic stem cells (HSCs) in the bone marrow. Under the influence of chemokines, OCPs enter circulation and migrate to bone tissue at resorption sites [17]. OCPs require two essential cytokines to differentiate into osteoclasts, macrophage colony-stimulating factor (M-CSF) and receptor activator of NF kappa B ligand (RANKL). M-CSF, produced by marrow stromal cells, is responsible for the proliferation, survival, and differentiation of OCPs [16]. RANKL, mainly secreted by osteocytes, primes OCPs differentiating into osteoclasts and activates mature osteoclasts [17,18,19]. The detailed signaling pathway and regulatory mechanisms governing osteoclastogenesis are described below. Figure 1 depicts the underlying mechanisms of osteoclast differentiation and the regulation of bone resorption, including the RANKL/RANK/OPG and IL-1/TNF-α signaling pathways.

#### 3.4.1. RANKL/RANK/OPG Signaling Pathway

The RANKL/RANK/OPG signaling pathway is one of the most extensively studied pathways in bone homeostasis. After RANKL binds to RANK on osteoclasts, tumor necrosis factor receptor-related factor-6 (TRAF-6) and mitogen-activated protein kinase (MAPK) pathways are activated. TRAF-6 transmits signals through inhibitor of κB kinase (IκK), which subsequently activates NF-κB-induced kinase (NIK), thereby regulating osteoclast maturation, differentiation, or apoptosis. TRAF-6 also transmits signals through c-Src, phosphatidylinositol 3-kinase (PI3K), and protein kinase B (PKB, Akt), ultimately regulating osteoclast differentiation. The MAPK pathway transduces signals through extracellular signal-regulated kinase (ERK1/2), c-Jun N-terminal kinase (JNK), and P38MAPK, leading to the activation of transcription factors such as c-Fos, activator protein-1 (AP-1), and nuclear factor of activated T cells-1 (NFATc1). NFATc1, a master transcription factor, stimulates the expression of osteoclastogenic genes and promotes osteoclast differentiation [13,17,18].

To regulate this pathway, osteoprotegerin (OPG), a member of the TNF receptor family mainly produced by osteoblasts, binds to RANKL and prevents its interaction with RANK. Thus, osteoclast activity depends, at least in part, on the relative balance between RANKL and OPG [20].

There are additional factors that modulate the RANKL/RANK/OPG signaling pathway. Mucosa-associated lymphoid tissue lymphoma translocation factor 1 (MALT1) has been shown to directly regulate NF-κB activation and, consequently, the expression of NFATc1. Moreover, the recently identified receptor leucine-rich G protein-coupled receptor 4 (LGR4) has been demonstrated to competitively bind to RANKL [17].

#### 3.4.2. IL-1/TNF-α Signaling Pathway

Interleukin-1 (IL-1) can induce tumor necrosis factor-α (TNF-α) to promote the differentiation of OCPs into osteoclasts through two distinct mechanisms. First, TNF-α acts on osteoblasts to stimulate the production of M-CSF and IL-6, thereby indirectly enhancing OCP differentiation. Second, TNF-α can directly bind to OCPs and facilitate their differentiation via activation of the NF-κB, JNK, p38, or ERK signaling pathways [17].

### 3.5. Osteoblast Differentiation and Proliferation

**Figure 2:**
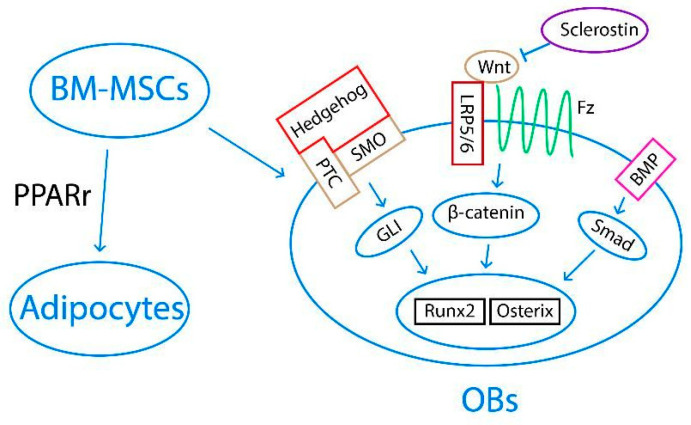
The detailed mechanisms of osteoblast differentiation and proliferation, including the Wnt/β-catenin signaling pathway, BMP–Smad signaling pathway, hedgehog signaling pathway, and pathways affected by other related factors and regulators. BM-MSCs: bone marrow mesenchymal stem cells; PPARγ: peroxisome proliferator-activated receptor γ; LRP: low-density lipoprotein receptor-related protein; Fz: Frizzled; BMPs: bone morphogenetic proteins; Runx2: Runt-related transcription factor 2; OBs: osteoblasts.

Osteoblasts are the principal functional cells responsible for bone formation. They are derived from precursor osteoblasts that originate from multipotent bone marrow mesenchymal stem cells (BM-MSCs). After maturation, osteoblasts reside on the bone surfaces and facilitate bone formation. They can also become embedded in the bone matrix and subsequently differentiate into osteocytes. Like osteoclasts, osteoblast differentiation and proliferation are regulated by several prominent signaling pathways. Several key transcription factors are responsible for osteoblastogenesis. Osterix and Runt-related transcription factor 2 (Runx2) direct BM-MSC differentiation into osteoblasts, whereas peroxisome proliferator-activated receptor γ (PPARγ) drives BM-MSC differentiation into adipocytes [13,17,18]. Cell-to-cell communication through exosomal exchange is also believed to influence the lineage commitment of BM-MSCs [21]. Moreover, several well-known signaling pathways influence osteoblastogenesis. Figure 2 illustrates the mechanisms of osteoblast differentiation and proliferation, including the Wnt/β-catenin, BMP–Smad, and Hedgehog signaling pathways, as well as pathways affected by other related factors and regulators.

#### 3.5.1. Wnt/β-Catenin Signaling Pathway

The Wnt signaling pathway is activated through binding of Wnt protein to low-density lipoprotein receptor-related proteins (LRP5/6) and Frizzled (Fz) receptors, located on the osteoblast membrane. LRP5/6 and Fz promote the stabilization of intracellular β-catenin. Stabilized β-catenin then translocates into the nucleus and regulates the expression of Osterix and Runx2. In contrast, sclerostin secreted by osteocytes inhibits Wnt binding to osteoblast receptors [17]. An animal study in 2019 found that the SOST gene, which is expressed almost exclusively by osteocytes and encodes the protein sclerostin, is also expressed by a variety of non-osteocyte cells. This finding challenges current paradigms of osteocyte exclusivity in bone modeling regulation [22].

#### 3.5.2. BMP–Smad Signaling Pathway

Bone morphogenetic proteins (BMPs) bind to specific receptors on the osteoblast membrane to phosphorylate downstream Smad proteins. Following phosphorylation, Smad proteins activate transcription factors, including Runx2 and Osterix, thereby facilitating osteoblast differentiation and proliferation [17].

#### 3.5.3. Hedgehog Signaling Pathway

The Hedgehog (Hh) signaling pathway begins with the binding of Hh ligands to the patched PTC and SMO receptors on mesenchymal stem cells (MSCs). This interaction leads to the activation of GLI transcription factors, which subsequently translocate into the nucleus and promote Runx2 expression. As a result, MSCs differentiate into osteoblasts rather than adipocytes [17].

As a bone micro-fracture needs remodeling, the osteocytes recruit HSCs. First of all, the signal from RANKL binding to RANK on OCPs and the direct/indirect effect of TNF-α on OCPs helps with differentiation into osteoclasts. Osteoclasts resorb the damaged bone. After resorption, MSCs are recruited and differentiate into osteoblasts through the Wnt, BMP, and Hh pathways. Runx2, an important transcription factor, is activated and leads to subsequent osteogenesis. Apart from the direct osteogenesis effect of osteoblasts, BM-MSCs per se may enhance bone repair by secreting paracrine factors that recruit blood vessels and the accompanying perivascular stem cells [23].

#### 3.5.4. Other Related Factors and Regulators

Other regulators of the osteogenic process include NOTCH cascades, Cdc42, Sox9 and Msx2 genes, histone deacetylases (HDACs), fibroblast growth factor (FGF), and parathyroid hormone-related peptide (PTHrP) cytokines [13,24]. Whether the NOTCH pathway promotes or inhibits osteoclastogenesis remains under debate [17]. Cdc42, activated by M-CSF and RANKL, has also been shown to influence osteoclast function by modulating M-CSF-stimulated cyclin D expression and phosphorylation of Rb, as well as inducing caspase 3 and Bim, thereby contributing to osteoclast proliferation and apoptosis [25].

Several additional factors influence osteogenesis. Icariin, an extract essence derived from the traditional Chinese medicinal plant Epimedium, has been shown to enhance osteogenic differentiation of osteoblasts and inhibit osteoclast differentiation in mouse cell studies [26]. Melatonin increases Runx2 expression and decreases PPARγ expression in MSCs, thereby promoting their differentiation into osteoblasts [27]. Adrenarche, the physiological increase in adrenal androgen secretion, may also contribute to improved bone status. A study enrolling 205 adolescents revealed that adrenarche is one determinant of bone mineral status in children [28].

Glucocorticoid inhibits bone formation through actions on both osteoclasts and osteoblasts. For osteoclasts, glucocorticoids increase the expression of RANKL and colony-stimulating factor-1 and reduces OPG expression, leading to enhanced osteoclastogenesis. In osteoblasts, glucocorticoids direct MSC differentiation away from the osteoblasts lineage and increase apoptosis of mature osteoblasts. They also impair osteoblast function through insulin-like growth factor 1 (IGF-1) signaling [29]. In addition, hypoxic conditions can enhance osteoclastogenesis and increase the ability of osteoclasts. Hypoxia can also inhibit osteoblast differentiation by affecting the Runx2, Sox9, Wnt, and PI3K/Akt signaling pathways, leading to reduced bone formation [14].

Interferon-gamma (IFN-γ) is thought to be chronically elevated in patients with osteoporosis. It promotes osteoclast formation through transcriptional stimulation of mini-TrpRS [30]. Fibroblast growth factor (FGF) can expand bone marrow mesenchymal, hematopoietic stem, and progenitor cells and further enhance osteoblastogenesis and osteoclastogenesis [31]. Histone acetyltransferases promote osteoblastogenesis and osteoclastogenesis, whereas HDACs inhibit the differentiation of osteoblasts and osteoclasts [18].

## 4. Diabetes Mellitus-Related Osteoporosis (DOP): Cellular and Molecular Mechanisms of Osteoporosis

With the continuous aging of the global population and changes in lifestyle and dietary habits, diabetes mellitus has become a major public health issue worldwide. Globally, approximately 537 million people are affected by diabetes, making it the third most prevalent non-communicable disease, following cardiovascular diseases and cancer [32]. Prolonged abnormal blood glucose levels can lead to microvascular complications, such as diabetic retinopathy and nephropathy; macrovascular complications, including coronary artery disease, heart failure, and peripheral artery disease; neuropathy; and diabetic foot. Diabetes also disrupts bone metabolism and contributes to the development of osteoporosis [1].

Studies on type 2 diabetes mellitus (T2DM) have reported inconsistent findings regarding bone mineral density (BMD). Several studies have demonstrated that individuals with T2DM exhibit higher BMD than age-matched non-diabetic controls, particularly during the early stages of the disease [1,33]. This paradox primarily arises from the coexistence of relatively preserved or even increased BMD and an elevated fracture risk in diabetes-related osteoporosis. First, insulin is an anabolic hormone, and patients with T2DM often exhibit normal or elevated circulating insulin levels, which can stimulate bone formation and contribute to higher BMD. In addition, patients with T2DM typically have a higher body mass index (BMI), resulting in greater mechanical loading on the skeleton and, consequently, higher Dual-energy X-ray absorptiometry-measured BMD. In contrast, the increased fracture risk in DOP is largely attributable to impaired bone quality rather than bone quantity. Chronic hyperglycemia promotes alterations in bone biomechanics and the accumulation of advanced glycation end products, which compromise bone material properties. Furthermore, patients with diabetes have an increased risk of falls due to hypoglycemic episodes, visual impairment from diabetic retinopathy, and postural instability related to neuropathy. A higher BMI may also increase the traumatic load during falls, further contributing to fracture risk. Collectively, these mechanisms explain the paradoxical coexistence of higher BMD and increased fracture susceptibility in DOP [8]. However, longitudinal evidence indicates that this apparent skeletal advantage is not sustained; one study showed that although individuals without T2DM initially had lower bone mass than those with T2DM, prolonged diabetes duration was associated with a progressive decline in BMD, accompanied by a markedly increased prevalence of osteoporosis and fracture risk in the diabetic population [34]. Consistently, a meta-analysis of 21 studies involving 11,603 patients with T2DM reported an overall osteoporosis prevalence of 27.67% [35]. Importantly, despite preserved or even increased BMD in early-stage T2DM, fracture risk is approximately twofold higher than in the general population, largely reflecting impaired bone quality rather than reduced bone quantity [36].

In contrast, type 1 diabetes mellitus (T1DM) is associated with a substantially higher fracture risk. Patients with T1DM have an estimated sixfold increase in fracture risk compared with healthy individuals [36]. Bone complications in T1DM are generally more severe than those in T2DM, largely due to the absence of insulin’s anabolic effects on bone [37,38].

Diabetes affects not only BMD but also bone quality and microarchitectural integrity. Fracture risk has been shown to correlate imperfectly with BMD, as many fractures occur in individuals whose T-scores do not meet the diagnostic threshold for osteoporosis (T-score < −2.5) [39]. Moreover, a synergistic effect appears to exist between diabetes and osteoporosis, as patients with both conditions have a significantly increased risk of all-cause mortality [40]. Despite the close relationship between diabetes and osteoporosis, patients with T2DM often exhibit limited awareness of osteoporosis and bone-protective lifestyle behaviors [41]. According to the Asia Pacific Osteoporosis Coalition, only 31% of patients with diabetes have undergone bone health assessments [1].

**Figure 3:**
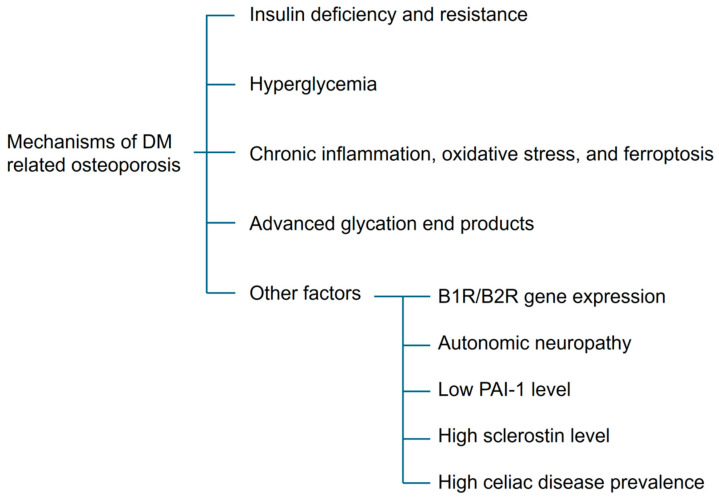
A brief summary of the mechanisms of diabetes mellitus-related osteoporosis (DOP).

Overall, diabetic osteoporosis (DOP) is a metabolic bone disorder characterized by impaired bone structure, reduced bone strength, and increased fracture risk resulting from hyperglycemia and insulin resistance [42]. Its pathogenesis is multifactorial and involves vascular dysfunction, chronic inflammation, oxidative stress, hyperglycemia, and insulin deficiency or resistance [32]. The following sections outline the major cellular and molecular mechanisms underlying DOP, as summarized in Figure 3.

### 4.1. Mechanism

The molecular mechanisms of DOP can be broadly categorized into endocrine dysregulation, metabolic toxicity, oxidative stress, and impaired bone–cell signaling.

#### 4.1.1. Insulin Deficiency and Resistance

Insulin plays a critical role in maintaining bone health by directly stimulating osteoblast activity and indirectly promoting RUNX2 transcription [43]. Insulin-like growth factor 1 (IGF-1) also supports bone formation. Insulin suppresses the expression of IGF-binding protein 1 (IGFBP-1) in osteoblasts; thus, insulin deficiency leads to elevated IGFBP-1 levels, reduced bioavailability of free IGF-1, and impaired bone formation [42].

#### 4.1.2. Hyperglycemia

Insulin resistance ultimately leads to chronic hyperglycemia, which adversely affects bone through multiple mechanisms. A high-glucose environment enhances glycolysis and promotes osteoclast differentiation [42]. Hyperglycemia also destabilizes hypoxia-inducible factor 1α (HIF-1α) by promoting its ubiquitination and proteasomal degradation, thereby reducing its inhibitory effect on osteoclastogenesis [42].

Hyperglycemia suppresses RUNX2 expression and inhibits osteoblast differentiation [43]. Glucose also promotes differentiation of BM-MSCs into adipocytes through non-canonical Wnt/protein kinase C pathway [42]. Animal studies have demonstrated lipid accumulation in bone marrow and thinning of cortical bone under diabetic conditions [44].

Hyperglycemia can directly damage osteoblasts through hyperosmolar stress [43]. Osteocalcin, a hormone secreted by osteoblasts, promotes insulin secretion and bone mineralization [45]. Serum osteocalcin levels are inversely correlated with blood glucose levels in patients with T2DM, suggesting a bidirectional relationship between hyperglycemia and bone dysfunction [42].

Hyperglycemia also indirectly impairs bone health. Osmotic diuresis reduces renal calcium reabsorption, leading to hypocalcemia and secondary hyperparathyroidism, which enhances bone resorption [42]. In addition, disrupted lipid metabolism increases very-low-density lipoprotein and total cholesterol levels, contributing to vascular dysfunction. Impaired bone perfusion compromises bone integrity and increases susceptibility to microdamage [45]. Diabetic nephropathy further reduces vitamin D synthesis and calcium absorption, exacerbating bone loss [1].

#### 4.1.3. Chronic Inflammation, Oxidative Stress, and Ferroptosis

Hyperglycemia induces excessive production of reactive oxygen species (ROS), thereby disrupting redox homeostasis and contributing to chronic low-grade inflammation in diabetic bone [42,43]. Elevated ROS levels enhance osteoclast differentiation through activation of the MAPK and NF-κB signaling pathways and induce apoptosis of BM-MSCs and osteoblasts [32,42,46]. In addition to apoptosis, hyperglycemia promotes ferroptosis, an iron-dependent form of regulated cell death characterized by lipid peroxidation and ROS accumulation. Under hyperglycemic conditions, intracellular Fe^2+^ catalyzes lipid oxidation via the Fenton reaction, further amplifying oxidative damage. Mechanistically, hyperglycemia induces ferroptosis in osteoblasts through activation of the METTL3/ASK1–p38 signaling pathway and dysregulation of ferroptosis-related genes, including SLC7A11 and GPX4 [42,46]. Collectively, oxidative stress and ferroptosis-mediated cellular dysfunction contribute to impaired bone formation and enhanced bone resorption in diabetes-related osteoporosis.

In diabetes-related osteoporosis, inflammation is typically characterized by a state of low-grade, metabolically driven chronic inflammation that primarily impairs osteoblast survival and function through oxidative stress and NF-κB-dependent signaling. In contrast, estrogen deficiency osteoporosis is associated with immune cell activation, particularly involving T cells and macrophages, which promotes excessive osteoclastogenesis and a high-turnover, resorption-dominant bone remodeling state.

#### 4.1.4. Advanced Glycation End Products (AGEs)

Hyperglycemia increases levels of reactive α-oxoaldehydes, including glyoxal, methylglyoxal, and 3-deoxyglucosone, which react with proteins to form advanced glycation end products (AGEs) via the Maillard reaction [38,42]. AGEs impair bone quality through both structural and cellular mechanisms.

Structurally, AGEs accumulate in bone collagen, reducing elasticity and weakening the bone’s ability to absorb mechanical stress [1,45,47,48]. At the cellular level, AGE–receptor for AGE (RAGE) interactions promote osteoclastogenesis by upregulating RANKL expression and impair osteoblast proliferation, differentiation, and survival by inhibiting PI3K, ERK, and Wnt signaling pathways [38,42,45]. AGE–RAGE signaling also increases transforming growth factor-β expression and suppresses stromal cell mineralization [32].

#### 4.1.5. Other Mechanisms

Additional mechanisms involved in DOP are currently being investigated. Activation of β3-adrenergic receptors has been shown to counteract bone resorption in animal models, although the relationship between diabetic autonomic neuropathy and bone remodeling remains unclear [44]. Gene deletion of bradykinin receptors B1 and B2 in mice results in severe diabetic complications and marked bone loss, suggesting a potential role for these receptors in DOP [44].

Plasminogen activator inhibitor-1 (PAI-1) deficiency has been shown to reduce bone loss by inhibiting osteoclast activity; however, PAI-1 also promotes adipogenesis and suppresses osteoblast function. Interestingly, patients with T2DM and osteoporosis exhibit lower circulating PAI-1 levels than those without osteoporosis [45].

Sclerostin, an osteocyte-derived inhibitor of Wnt signaling, suppresses osteoblast activity and enhances RANKL-mediated osteoclast activation. Elevated serum sclerostin levels have been reported in both T1DM and T2DM and are negatively regulated by mechanical loading [32,47].

In diabetes-related osteoporosis, elevated sclerostin levels are closely associated with hyperglycemia, mechanical unloading, and AGE-mediated osteocyte dysfunction, leading to suppression of Wnt/β-catenin signaling and impaired osteoblast activity. In contrast, in estrogen deficiency osteoporosis, sclerostin upregulation is primarily driven by estrogen withdrawal, representing a key mechanism underlying reduced bone formation.

T1DM is frequently associated with autoimmune comorbidities such as celiac disease, which contributes to osteoporosis through malabsorption, vitamin D deficiency, and low body weight [43].

Table 1 provides a comparative chart between diabetes-related osteoporosis (DOP) and estrogen deficiency osteoporosis (EDOP). In brief, DOP is primarily driven by metabolic toxicity, oxidative stress, ferroptosis, and AGE-mediated impairment of bone quality and mechanotransduction, whereas EDOP is characterized by estrogen receptor signaling loss, immune activation, and RANKL-dependent high-turnover bone resorption.

### 4.2. Diagnostic Tools

The high prevalence of osteoporosis among patients with diabetes mellitus underscores the importance of accurate and timely diagnosis. Several diagnostic tools are currently used to assess bone mass, bone quality, and fracture risk in this population.

#### 4.2.1. Dual-Energy X-Ray Absorptiometry (DXA)

Dual-energy X-ray absorptiometry (DXA) is considered the gold standard for measuring BMD, providing quantitative assessments at clinically relevant sites such as the lumbar spine and hip [32]. However, these commonly evaluated regions are susceptible to artifacts caused by lumbar spine degeneration and abdominal aortic calcification, which may compromise measurement accuracy [47].

As discussed previously, patients with T2DM, particularly in the early stages of disease, may exhibit normal or elevated BMD despite an increased risk of fractures. This observation highlights a major limitation of DXA, as BMD reflects bone quantity rather than bone quality. Impairments in bone microarchitecture and material properties contribute substantially to fracture risk and may occur independently of BMD values [32,37,49,50].

Nevertheless, studies have consistently demonstrated an association between T1DM, reduced BMD, and an increased risk of osteoporosis at specific skeletal sites, particularly the femoral neck and lumbar spine [51].

#### 4.2.2. Fracture Risk Assessment (FRAX)

The Fracture Risk Assessment Tool (FRAX), developed by the World Health Organization, estimates the 10-year probability of major osteoporotic and hip fractures based on clinical risk factors, including age, sex, body mass index, prior fracture history, parental hip fracture, smoking, glucocorticoid use, rheumatoid arthritis, secondary osteoporosis, and alcohol consumption. However, because type 2 diabetes mellitus is not incorporated as a secondary cause of osteoporosis in the FRAX algorithm, fracture risk may be underestimated in this population [32,37]. Moreover, FRAX was designed as a risk prediction tool for treatment-naïve individuals and is therefore not applicable to patients with established osteoporosis, prior fragility fractures, or ongoing anti-osteoporosis therapy. Nevertheless, FRAX scores generally demonstrate comparable fracture risk trends among individuals with and without diabetes [47].

#### 4.2.3. Trabecular Bone Score (TBS)

Trabecular bone score (TBS) is a noninvasive, texture-based parameter derived from DXA images that provides indirect information on trabecular bone microarchitecture, including trabecular number, density, and separation [50]. Higher TBS values indicate better trabecular integrity and greater resistance to fracture.

Clinical studies have demonstrated that TBS is useful in evaluating fracture risk in various forms of secondary osteoporosis, including hyperparathyroidism and glucocorticoid-induced osteoporosis [37]. Patients with T2DM generally exhibit lower TBS values than individuals with a normal glycemic status, and TBS may be a more sensitive predictor of fracture risk than BMD alone in this population [50].

#### 4.2.4. High-Resolution Peripheral Quantitative Computed Tomography (HR-pQCT)

High-resolution peripheral quantitative computed tomography (HR-pQCT) is a noninvasive imaging technique that enables detailed assessment of bone microarchitecture, including volumetric BMD (vBMD) and trabecular and cortical structural parameters [32]. This modality allows for separate evaluation of cortical and trabecular bone compartments and provides three-dimensional structural information.

This modality is commonly applied to peripheral skeletal sites, particularly the distal radius and distal tibia, for fracture risk evaluation. Several studies have reported that HR-pQCT is more sensitive than DXA in detecting treatment-related or age-related changes in bone density and microstructure. Cross-sectional studies have demonstrated that patients with type 1 diabetes mellitus exhibit reduced cortical thickness and decreased cortical vBMD at the ultra-distal tibia, highlighting the utility of HR-pQCT in detecting diabetes-associated cortical bone deficits that are not captured by areal BMD measurements [47,50].

#### 4.2.5. Bone Histomorphometry

Bone histomorphometry involves the quantitative analysis of bone microstructure using two-dimensional microscopic images obtained from bone biopsies. This technique provides detailed information on parameters such as trabecular thickness, connectivity, bone formation rate, cortical thickness, and porosity. However, its clinical application in patients with diabetes is limited due to its invasive nature and the inability of DXA to differentiate trabecular from cortical bone proportions. Currently, bone histomorphometry is frequently utilized in the investigation and creation of medications for osteoporosis prevention and treatment [47].

#### 4.2.6. Microindentation

Microindentation techniques, particularly reference point indentation (RPI), have been proposed as methods for directly assessing bone material strength. Using devices such as the OsteoProbe, the bone material strength index (BMSi) can be measured in vivo. Microindentation may complement BMD measurements by providing insight into bone mechanical properties; however, current evidence regarding the association between BMSi and fracture risk remains inconsistent [47].

Clinically, DXA and FRAX serve as appropriate initial tools for assessing bone quantity. When bone mineral density and bone quality appear discordant, adjunctive modalities such as trabecular bone score and high-resolution peripheral quantitative computed tomography should be considered for further evaluation. In contrast, techniques such as bone histomorphometry and microindentation have limited accessibility, high costs, and are currently used primarily in research settings rather than routine clinical practice.

### 4.3. Management

The management of diabetic osteoporosis should be comprehensive and include lifestyle modification, nutritional support, and pharmacological therapy. Particular attention should be paid to glycemic control, which is of paramount importance in mitigating the deleterious effects of diabetes on bone health.

In patients with T1DM, the primary defect contributing to osteoporosis is impaired osteoblastic function rather than excessive osteoclastic activity. Therefore, anabolic therapies, such as parathyroid hormone (PTH) analogs, may theoretically be more effective than antiresorptive agents in this population [43]. In contrast, patients with T2DM may benefit from both anabolic and antiresorptive therapies, depending on disease stage and individual fracture risk.

With respect to glucose-lowering medications, metformin and sulfonylureas are generally regarded as neutral in terms of fracture risk; however, experimental evidence suggests that metformin exerts pro-osteoblastic effects via AMPK–RUNX2 signaling, improves insulin sensitivity, and reduces oxidative stress through activation of the Keap1/Nrf2/HO-1 signaling pathway, thereby exerting protective effects on bone microarchitecture [42]. Meanwhile, sulfonylureas such as glimepiride may enhance osteoblast differentiation under hyperglycemic or estrogen-deficient conditions. The skeletal effects of incretin-based therapies, including GLP-1 receptor agonists and DPP-4 inhibitors, remain inconclusive, with animal studies suggesting potential benefits on trabecular bone. In contrast, thiazolidinediones are consistently associated with bone loss and increased fracture risk through PPARγ-mediated adipogenesis, osteoclast activation, and upregulation of osteocyte-derived sclerostin and RANKL. Data on SGLT2 inhibitors are limited and conflicting, although canagliflozin has been reported to exacerbate trabecular bone loss in experimental models [52].

Loop diuretics should be avoided in patients with diabetic nephropathy, as they increase renal calcium excretion and may exacerbate bone loss [43]. For all glucose-lowering therapies, caution is warranted to prevent hypoglycemic episodes, which increase the risk of falls and subsequent fractures [50,53].

Several osteoporosis-specific pharmacological agents have demonstrated efficacy in patients with diabetes. Bisphosphonates, androgens, and receptor activator of nuclear factor κB ligand (RANKL) inhibitors are among the most beneficial treatment options for diabetic osteoporosis [45]. Notably, in adults older than 45 years with osteoporosis, denosumab has been shown to reduce the incidence of diabetic osteoporosis more effectively than oral bisphosphonates [54].

Exosomal signaling plays a critical role in regulating BM-MSC differentiation and the progression of osteoporosis. Mesenchymal stem cell-derived exosomes (MSC-Exos) act as cell-free mediators of intercellular communication by delivering bioactive cargoes, including microRNAs, proteins, and signaling molecules, to BM-MSCs within the bone microenvironment. Table 2 summarizes how exosomal signaling influences different cell types. Exosomal signaling not only promotes osteoblast differentiation while suppressing adipogenic lineage commitment—thereby restoring the imbalance between bone formation and resorption characteristic of osteoporosis—but also enhances BM-MSC survival, angiogenic–osteogenic coupling, and immunomodulation, collectively creating a microenvironment conducive to bone regeneration [55]. In brief, MSC-derived exosomes restore bone homeostasis by coordinately regulating BM-MSC osteogenic differentiation, osteoblast survival, osteocyte signaling, angiogenesis, and osteoimmune balance.

Recent studies have also shown that adipose-derived mesenchymal stem cells (AD-MSCs) can alleviate diabetic osteoporosis by suppressing NLRP3 inflammasome activation in osteoclasts in rat models. Although current evidence is limited to cellular and animal studies, this strategy represents a promising therapeutic approach for the treatment of diabetic osteoporosis [56].

Finally, the elevated fracture risk observed in patients with diabetes is attributable not only to impaired bone mass and quality but also to an increased propensity for falls. Diabetes-related complications, including peripheral neuropathy, visual impairment, and adverse effects of certain medications, substantially contribute to fall risk and should be systematically addressed as part of a comprehensive management strategy [43,50].

## 5. Estrogen Deficiency-Induced Osteoporosis: Cellular and Molecular Mechanisms of Osteoporosis

Estrogen has long been recognized as a critical protective factor against osteoporosis. The increased susceptibility to osteoporosis observed in postmenopausal women is primarily attributable to estrogen deficiency. One study reported that BMD was normal in the majority (64.4%) of women younger than 55 years; however, the prevalence of osteoporosis approximately doubled every five years thereafter, reaching 50.3% in women aged 85 years and older [5]. According to the International Osteoporosis Foundation, up to half of all women and one in five men aged 50 years or older will experience a fragility fracture during their lifetime. In addition to natural menopause, the incidence of iatrogenic menopause has increased as a result of comprehensive oncological treatments, including surgical, pharmacological, and radiological interventions [57]. Compared with natural menopause, surgical menopause is associated with more severe climacteric symptoms and a higher risk of osteoporosis [58].

Although estrogen plays a crucial role in skeletal homeostasis in both women and men, women are more vulnerable to osteoporotic fractures because of the abrupt decline in estrogen levels after menopause. Nevertheless, men experience higher mortality rates following osteoporotic fractures [3].

Sex hormone deficiency, particularly estrogen deficiency, is a primary contributor to age-related osteoporosis. Cumulative lifetime exposure to endogenous estrogen appears to exert protective effects against postmenopausal osteoporosis (PMOP) [59]. The association between oophorectomy before natural menopause and reduced BMD further supports this concept [60]. Fistarol et al. demonstrated that time since menopause, rather than chronological age, is the principal determinant of PMOP [61]. Furthermore, premature ovarian insufficiency leading to early-onset menopause is associated with lower postmenopausal BMD and an increased risk of fragility fractures [59,62,63].

### 5.1. Mechanism

Reduced serum estrogen levels disrupt bone homeostasis through multiple molecular and cellular pathways. Estrogen exerts its biological effects via three principal receptors: estrogen receptor alpha (ERα), estrogen receptor beta (ERβ), and the G-protein-coupled estrogen receptor (GPER). ERα and ERβ are expressed in osteoblasts, osteoclasts, osteocytes, and BM-MSCs and primarily mediate genomic estrogen signaling, whereas GPER mediates rapid non-genomic signaling.

#### 5.1.1. The Genomic Pathway

In osteoblasts, estrogen deficiency suppresses osteogenesis by downregulating the Wnt/β-catenin and BMP–Smad signaling pathways. Under estrogen-deficient conditions, BM-MSCs preferentially differentiate into adipocytes rather than osteoblasts, thereby impairing bone formation [64,65]. Estradiol (E2), the most biologically active estrogen, normally suppresses receptor activator of nuclear factor κB ligand (RANKL) expression and enhances osteoprotegerin (OPG) production in bone-lining cells. Estrogen deficiency disrupts this balance, leading to increased osteoclast activity and bone resorption [3].

Estrogen receptor 1 (ESR1), which encodes ERα, participates in the ESR1–Keap1–Nrf2 signaling axis, thereby reducing oxidative stress and supporting osteoblast function. Declining estrogen levels increase oxidative stress, further compromising bone formation [2].

In osteoclasts, estrogen deficiency enhances RANKL-mediated signaling by reducing the inhibitory effects of estrogen and its receptors [64]. Reduced estrogen levels are also associated with decreased transforming growth factor beta (TGF-β), which normally limits osteoclast differentiation and promotes osteoclast apoptosis [65]. In addition, estrogen deficiency stimulates circulating macrophages to secrete proinflammatory cytokines, including interleukin-1, interleukin-4, interleukin-6, and tumor necrosis factor alpha, which further increase RANKL expression and accelerate osteoclastogenesis, resulting in trabecular bone loss [3].

Osteocytes also contribute to estrogen deficiency-induced bone loss by increasing secretion of sclerostin, an inhibitor of the Wnt/β-catenin signaling pathway. Elevated sclerostin levels suppress osteoblast activity and impair bone formation [65].

#### 5.1.2. The Non-Genomic Pathway

The non-genomic pathway involves rapid signaling events initiated at the cell membrane. Estrogen binding to GPER activates the cyclic adenosine monophosphate/protein kinase A/cAMP response element-binding protein signaling cascade, thereby promoting BM-MSC proliferation [3].

Beyond direct receptor-mediated effects, estrogen also enhances bone formation through vascular and hormonal modulation. Estrogen signaling interacts with the phosphoinositide 3-kinase/protein kinase B pathway, leading to increased endothelial nitric oxide synthase activity and angiogenesis. Improved bone microcirculation supports osteogenesis and skeletal integrity [2]. In addition, estrogen deficiency is associated with upregulation of the renin–angiotensin–aldosterone system. Activation of the angiotensin-converting enzyme/angiotensin II/angiotensin II type 1 receptor axis promotes osteoclastogenesis by altering the OPG/RANKL ratio and suppresses osteoblast differentiation by inhibiting Wnt/β-catenin signaling [64].

#### 5.1.3. The Estrogen Receptor (ER)-Related Mechanisms of Osteoporosis

Estrogen regulates the expression of its own receptors, and estrogen deficiency is associated with reduced estrogen receptor expression in bone marrow cells [64].

In addition to ligand-dependent mechanisms, estrogen receptors can be activated through ligand-independent pathways that do not require estrogen binding. These pathways typically involve phosphorylation by kinases such as protein kinase A, protein kinase C, or components of the mitogen-activated protein kinase cascade. A well-characterized example is the osteogenic response to mechanical loading [3]. Osteocytes function as mechanosensors and translate mechanical stimuli into biochemical signals that activate estrogen receptors and maintain bone homeostasis [66].

Mechanical loading can activate ERα in a ligand-independent manner. Estrogen deficiency reduces ERα expression and impairs osteocyte responsiveness to mechanical stimuli, thereby compromising mechanotransduction and bone adaptation. Studies in female mice subjected to cortical loading have demonstrated minimal involvement of classical estrogen response element-mediated genomic signaling, further supporting the role of ligand-independent estrogen receptor activation in bone physiology [3].

#### 5.1.4. Ferroptosis

Interestingly, ferroptosis not only plays a critical role in DOP but also contributes to estrogen deficiency-induced osteoporosis. Clinical and experimental studies indicate that both iron deficiency and iron overload can adversely affect bone integrity, underscoring a context-dependent role of iron homeostasis in skeletal health. Ferroptosis has been implicated in RANKL-induced osteoclast differentiation, in which dysregulated iron metabolism and ferritinophagy promote osteoclastogenesis. Mechanistically, HIF-1α signaling appears to mediate ferritinophagy-driven ferroptosis in osteoclasts [67].

Overall, ferroptosis contributes to dysregulated bone metabolism in both DOP and PMOP; however, the underlying pathogenic contexts and predominant target cell populations differ. In DOP, ferroptosis is primarily driven by chronic hyperglycemia and metabolic stress, affecting osteoblasts, osteocytes, and bone marrow mesenchymal stem cells. In contrast, in PMOP, ferroptosis is closely associated with estrogen deficiency-related disturbances in iron metabolism and enhanced osteoclast activation.

### 5.2. Evaluation

General diagnostic tools for osteoporosis have been discussed previously. In the context of estrogen deficiency-induced osteoporosis, biochemical bone turnover markers provide additional insights into disease activity. Markers such as bone-specific alkaline phosphatase, osteocalcin, and hydroxyproline reflect osteoblast and osteoclast activity and are useful for monitoring disease progression and therapeutic response, although they are not diagnostic.

Camelia et al. reported an inverse correlation between lumbar spine BMD and bone turnover markers in postmenopausal women, with resorption markers increasing in proportion to the duration of estradiol deficiency [68].

Long non-coding RNAs, defined as transcripts longer than 200 nucleotides, regulate gene expression through transcriptional, post-transcriptional, and epigenetic mechanisms. The long non-coding RNA small-nucleolar RNA host gene 1 has been shown to be significantly downregulated in postmenopausal osteoporosis and to exhibit predictive value for disease presence. Plasma levels of this transcript increase following treatment, suggesting its potential utility as a biomarker for monitoring postmenopausal osteoporosis [69].

### 5.3. Management

According to U.S. Food and Drug Administration guidelines, the management of osteoporosis includes pharmacological therapy, dietary supplementation, and lifestyle modification. Six major classes of medications are approved for the treatment of osteoporosis: bisphosphonates, receptor activator of nuclear factor κB ligand inhibitors, parathyroid hormone analogs, sclerostin inhibitors, selective estrogen receptor modulators, and calcitonin analogs. Except for selective estrogen receptor modulators, which are prescribed exclusively for women, these agents may be used in both sexes.

Pharmacological treatment is recommended for postmenopausal women with osteoporosis or fragility fractures. In individuals with osteopenia, treatment should be considered when fracture risk assessment tools indicate a high probability of future fractures [70].

#### 5.3.1. Medications

**Figure 4:**
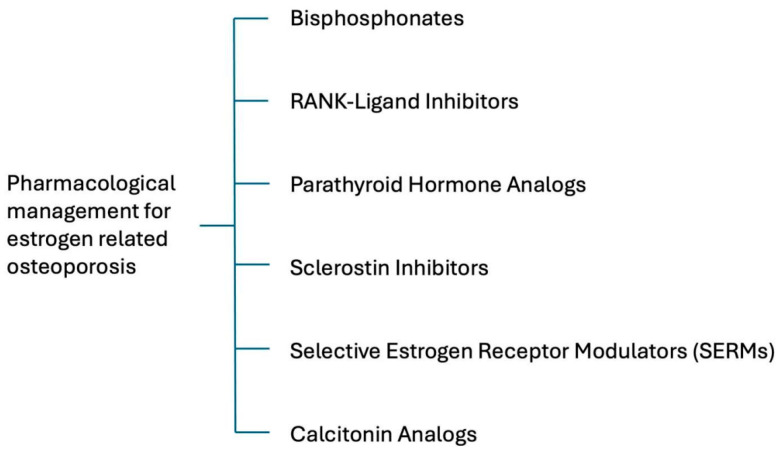
A brief summary of the pharmacological management of estrogen deficiency-induced osteoporosis.

Figure 4 briefs the pharmacological management of estrogen deficiency-induced osteoporosis (EDOP), including bisphosphonates, RANK-ligand (RANKL) inhibitors, PTH analogs, sclerostin Inhibitors, selective estrogen receptor modulators (SERMs), and calcitonin analogs.

##### Bisphosphonates

Alendronate, risedronate, and zoledronic acid are considered first-line agents for the treatment of postmenopausal osteoporosis. Clinical trials have demonstrated that bisphosphonates increase BMD at the hip and spine and reduce the risk of vertebral, hip, and wrist fractures. Common adverse effects include gastrointestinal issues, such as esophagitis and gastric ulcers [3,65]. In patients receiving long-term bisphosphonate therapy (typically more than 3 years, with a median duration of 7 years), excessive suppression of bone remodeling and a lack of regular bone turnover can lead to serious complications, including osteonecrosis of the jaw (ONJ) and atypical femoral fractures [17].

##### RANK-Ligand (RANKL) Inhibitors

Denosumab, a fully human IgG2 monoclonal antibody, mimics the action of osteoprotegerin (OPG) by inhibiting RANKL, thereby preventing osteoclast-mediated bone resorption [3]. Clinical studies have shown that denosumab is more effective than alendronate or ibandronate in reducing fracture risks, although it is comparable to zoledronate in this regard [71]. Due to its efficacy, denosumab is considered a first-line therapy for postmenopausal women at high fracture risk [8]. However, discontinuation of denosumab may result in osteoporotic fractures after a rapid decrease in BMD due to rebound osteoclast activity, a phenomenon sometimes referred to as a “drug holiday”. Similarly to bisphosphonates, long-term denosumab use is associated with an increased risk of mandibular osteonecrosis and atypical femoral fractures [17]. Denosumab is administered via subcutaneous injection every 6 months. Skin infections are among the most frequently reported adverse effects [65].

##### Parathyroid Hormone (PTH) Analogs

PTH analogs act by binding to the parathyroid hormone 1 receptor (PTH1R), activating transcription factors that promote an anabolic bone response. These agents stimulate osteoblast formation, enhance bone density, improve bone strength, and reduce the risk of fractures [72]. However, BMD loss (withdrawal effect) has been observed after discontinuation of PTH analogs [71]. PTH analogs include teriparatide and abaloparatide. In animal studies (e.g., in rats), these drugs have been associated with a risk of osteosarcoma, although this has not been confirmed in humans [3]. PTH analogs are generally reserved for patients with severe osteoporosis, those who are intolerant to other treatments, or individuals who continue to experience fractures despite antiresorptive therapy [65,70].

##### Sclerostin Inhibitors

Sclerostin, a protein secreted by osteocytes, inhibits bone formation by blocking the Wnt signaling pathway. Romosozumab, a monoclonal antibody targeting sclerostin, has been approved by the FDA in 2019 for the treatment of postmenopausal women with high fracture risk [17]. However, romosozumab has a limited period of effectiveness and therefore requires concomitant antiresorptive therapy [71]. Importantly, romosozumab use has been linked to an increased risk of adverse cardiovascular events. Therefore, it should be avoided in patients with a history of stroke or cardiovascular disease [3].

##### Selective Estrogen Receptor Modulators (SERMs)

Traditional hormone replacement therapy (HRT) using estrogen alone can lead to endometrial hyperplasia and endometrial carcinoma, unless the woman has undergone hysterectomy [65]. In such cases, progestogens are added to the regimen to mitigate this risk. However, the addition of progestogens may cause vaginal bleeding and further increase the risk of breast cancer when combined with estrogen [73]. Although HRT is considered a second-line option, it remains effective in preventing postmenopausal bone loss and reducing the risk of osteoporotic fracture [6].

Selective estrogen receptor modulators (SERMs) are a class of drugs that act on estrogen receptors, functioning as agonists or antagonists depending on the target tissue. Tamoxifen, a first-generation SERM, helps prevent osteoporosis by acting on osteoblasts and osteoclasts. However, its agonistic effect on the uterus raises the risk of endometrial hyperplasia or cancer [3]. Raloxifene is the first SERM approved for the treatment of postmenopausal osteoporosis and has been shown to reduce the risk of vertebral fractures while simultaneously offering protective effects against breast and endometrial cancers [65]. Bazedoxifene (BZA), a third-generation SERM, exhibits stronger antagonistic effects on estrogen receptors in the endometrium. The FDA has approved a combination product of BZA with conjugated equine estrogen (CEE), forming a tissue-selective estrogen complex (TSEC), for the treatment of menopausal vasomotor symptoms and postmenopausal osteoporosis. The TSEC relieves hot flashes, improves vulvo-vaginal atrophy and its symptoms, does not stimulate the endometrium, and prevents bone loss. It maintains the benefits of estrogen therapy without the effects associated with progestins [3,65,73,74,75].

##### Calcitonin Analogs

Calcitonin analogs, including salcatonin and carbocalcitonin (derived from salmon and eel), are used in the treatment of osteoporosis. Calcitonin regulates calcium metabolism and inhibits osteoclast activity by directly binding to osteoclast receptors. Exogenous calcitonin has been shown to improve BMD in patients with osteoporosis. In addition to its effects on BMD, calcitonin is also used to relieve bone pain caused by osteoporosis, primarily through activation of the endogenous opioid system [17]. Calcitonin is available in nasal spray or oral formulations. However, rare, serious hypersensitivity reactions—including bronchospasm, throat swelling, and anaphylactic shock—have been reported. With parenteral administration, nausea, abdominal discomfort, and a metallic taste are known side effects.

#### 5.3.2. Dietary Management

Adequate calcium and vitamin D intake is essential for the prevention of bone loss. Although calcium and vitamin D supplementation alone is insufficient to prevent fractures in individuals with osteoporosis, it serves as an important adjunct to pharmacologic interventions. For postmenopausal women, a total daily intake of 1200 mg of elemental calcium and 800 to 1000 IU of vitamin D is recommended [4].

Insufficient protein intake may negatively impact both muscle and bone mass, increasing the risk of osteoporotic fractures, especially in the elderly and postmenopausal women. In animal studies, protein restriction has been shown to result in a loss of muscle and bone mass; however, this effect can be mitigated through protein supplementation combined with hormone replacement therapy [76]. Although excessive protein was once believed to negatively affect bone health due to increased urinary calcium excretion, this effect is likely only significant when calcium intake is inadequate. Moreover, the acidic load from dietary protein appears to have a relatively minor impact on bone health [4,77].

With regard to other nutritional support, omega-3 fatty acids support calcium absorption and bone mineralization [77]. Extra virgin olive oil (EVOO) may have anti-osteoporotic properties and has been shown to improve spinal BMD in clinical studies [78]. Daily supplementation of sesame oil is found to prevent postmenopausal osteoporosis by maintaining serum estrogen and aromatase levels in an animal study [79]. Other dietary components that may provide bone health benefits, though currently lacking strong evidence, include vitamin C, vitamin K, magnesium, and isoflavones [3,4]. Certain substances, such as caffeine and excessive sodium intake, are associated with increased calcium excretion, which may adversely affect bone health [77].

#### 5.3.3. Lifestyle Modifications

##### Physical Activity and Exercise

Physical inactivity contributes to accelerated bone loss and reduced muscle strength, both of which increase the risk of osteoporotic fractures [3]. A study by Gang Chen et al. showed that longer daily sleep duration is associated with a higher risk of osteoporosis. The authors proposed that extended sleep might reflect lower levels of weight-bearing physical activity or could be secondary to chronic illness [80]. Engaging in weight-bearing and strength training exercises is beneficial for both bone formation and maintenance. Physical activity also helps reduce the risk of falls by improving muscle strength, agility, and balance [4]. A meta-analysis revealed that practicing Tai Chi (TC) can reduce the incidence of injurious falls by approximately 43–50% and improve postural stability [3]. However, exercises that involve high-impact, repetitive, or resisted trunk flexion should be avoided in patients with osteoporosis due to the increased risk of vertebral fractures [4].

##### Tobacco Use

Smoking is associated with a significant reduction in bone mass, potentially due to impaired calcium absorption and reduced 17β-estradiol levels. While the exact mechanisms are still under investigation, smoking is clearly linked to an increased risk of fractures and should be strictly avoided [3,4,65].

## 6. Discussion

Osteoporosis, often regarded as a “silent disease” is prevalent worldwide. If left untreated, it often results in fractures, which can lead to impaired quality of life and even increased mortality. Currently, BMD measured by DXA is considered the standard diagnostic tool for osteoporosis. However, since DM impairs not only bone mass but also bone microstructure, BMD alone may not fully reflect the overall bone condition. Therefore, a more comprehensive diagnostic and assessment approach should be explored.

A previous study found that T1DM may contribute to the development of osteoporosis by affecting plasma medium very-low-density lipoprotein cholesterol and increasing sex hormone-binding globulin levels. These mediators may serve as important biomarkers for the early detection and treatment of osteoporosis in patients with T1DM [81]. Another study demonstrated that by analyzing bone turnover markers using a support vector machine model, early detection and daily monitoring of osteoporosis in T2DM patients may be feasible [82].

Ferroptosis plays an important role both in DOP and EDOP. Emerging ferroptosis-associated therapies included lipid peroxidation inhibitors, iron chelators, and Nrf2/GPX4 activators in DOP or HIF-1α inhibitor in EDOP. Nevertheless, their clinical translation is limited by insufficient specificity, potential systemic effects on iron homeostasis, and a lack of long-term safety data. Moreover, disease-specific ferroptosis biomarkers and optimized delivery strategies remain major challenges for precision therapy [46,67].

Primary type I osteoporosis, which affects postmenopausal women globally, remains a significant public health concern. Although the time since menopause appears to be a stronger determinant of PMOP than age, serum estrogen levels seem to have limited impact on postmenopausal BMD [83]. It is generally believed that cumulative exposure to endogenous estrogen offers protective effects against PMOP. However, this concept appears to be less applicable when considering factors such as age at menarche (AAM) and prenatal estrogen exposure. One study showed that early menopause (between 40 and 45 years), rather than AAM, was associated with lower BMD in postmenopausal women. Another study found no association between osteoporosis susceptibility genes and AAM [59,84]. Furthermore, Samantha et al. found that longer menstruation duration was linked to an increased risk of PMOP in patients with prenatal exposure to diethylstilbestrol, challenging the traditional understanding. Further research is needed to clarify the relationship between lifetime estrogen exposure and PMOP [85].

Currently, the primary goal of ovarian cryopreservation is fertility preservation. However, a novel concept has been proposed to delay menopause and prevent PMOP by cryopreserving a portion of the abundant ovarian follicles during youth for later transplantation. This method could postpone menopause with only a minimal advancement of the natural menopausal onset [86].

Many treatments for gynecological cancers—such as hysterectomy plus bilateral salpingo-oophorectomy, radiotherapy, and chemotherapy—can result in the loss of ovarian function, leading to early menopause in women under the age of 45. As previously discussed, menstruation plays a key role in regulating bone homeostasis primarily through the action of estrogen. Better preservation of natural hormonal function may provide greater benefits for bone health. While hormone supplementation after menopause can improve BMD, whether menopausal hormone therapy should be administered in gynecologic cancer patients depends on the specific cancer type and the hormone sensitivity of the tumor cells [87].

Androgens play a fundamental role in skeletal development and maintenance through both direct and indirect mechanisms. Testosterone promotes bone modeling and remodeling by stimulating osteoblast proliferation and differentiation while suppressing osteoclast activity via androgen receptor (AR) signaling. In addition, testosterone serves as a critical precursor for estradiol through aromatization, and accumulating evidence indicates that estrogen derived from testosterone is the dominant regulator of bone resorption in men. Experimental and clinical studies demonstrate that low circulating estradiol, rather than testosterone alone, is strongly associated with increased bone turnover, impaired microarchitecture, and elevated fracture risk in aging men. Testosterone also contributes to periosteal bone expansion and cortical thickness, thereby influencing skeletal size and strength. Consequently, deficiencies in androgen action—whether due to hypogonadism or androgen deprivation therapy—are closely linked to osteoporosis and increased fracture susceptibility in men [3].

## 7. Conclusions

Osteoporosis is a highly prevalent disease worldwide, characterized by compromised bone strength and an increased risk of fractures. Diabetes mellitus affects bone structure through mechanisms such as insulin deficiency or resistance, hyperglycemia, chronic inflammation, oxidative stress, and the accumulation of advanced glycation end products. Estrogen exerts its effects through genomic, non-genomic, and ligand-independent signaling pathways. A decrease in estrogen levels can lead to a negative bone balance. The management of osteoporosis should be comprehensive, incorporating pharmacological interventions, lifestyle modifications, and dietary strategies. Future research should focus on novel therapeutic strategies, including exosome-based and ferroptosis-targeted therapies. In addition, artificial intelligence-assisted analysis of bone microarchitecture derived from HR-pQCT may provide improved approaches for evaluating skeletal integrity in DOP. However, their limitations should be considered and disclosed to patients with osteoporosis.

## Figures and Tables

**Table 1:** Comparisons of mechanisms between diabetes-related osteoporosis and estrogen deficiency osteoporosis.

Mechanistic Category	Diabetes-Related Osteoporosis (DOP)	Estrogen Deficiency Osteoporosis (EDOP)
Primary driver	Insulin deficiency/resistance and chronic hyperglycemia	Estrogen withdrawal
Dominant signaling disruption	Insulin/IGF-1, Wnt/β-catenin, PI3K–Akt	ERα/ERβ/GPER-mediated signaling
Oxidative stress origin	Hyperglycemia-induced ROS and mitochondrial dysfunction	Impaired ERα–Keap1–Nrf2 antioxidant defense
Inflammatory profile	Low-grade, metabolically driven chronic inflammation	Immune cell-driven inflammatory activation
Cell death mechanisms	Apoptosis and ferroptosis (METTL3/ASK1–p38)	Predominantly apoptosis
Matrix-related alterations	AGE-mediated collagen cross-linking and matrix stiffening	Minimal matrix glycation
Osteocyte–sclerostin axis	Metabolic osteocyte dysfunction with elevated sclerostin	Estrogen deficiency-induced osteocyte apoptosis and sclerostin upregulation
Mechanotransduction	Impaired due to AGE-stiffened matrix and reduced mechanosensitivity	Impaired due to loss of ligand-independent ERα activation
Bone phenotype	Variable BMD with reduced bone quality	Reduced BMD with high-turnover bone loss

**Table 2:** A summary of the influences of exosomal signaling on different cell types in bone remodeling.

Target Cell Type	Exosomal Signaling	Major Pathways Affected	Biological Effects Relevant to Osteoporosis
BM-MSCs	hiPSC-MSC–Exos; osteogenic cargos	Osteogenic transcriptional programs	↑ Osteogenic differentiation; ↑ RUNX2, ALP, COL1, OPN; enhanced bone formation
BM-MSCs	miR-375 (adipose MSC-Exos)	IGF signaling (IGFBP3 inhibition)	↑ ALP activity and mineralization; promotion of osteoblastic lineage commitment
BM-MSCs	miR-1263 (HUCMSC-Exos)	Hippo signaling (Mob1–YAP axis)	↓ Apoptosis; ↑ survival and regenerative capacity
Osteoblasts	MSC-Exos; miR-122-5p	MAPK/ERK signaling (SPRY2 inhibition)	↑ Proliferation and differentiation; ↑ OCN, OPN, BMP-2, RUNX2
Osteoblasts	Osteogenic miRNAs (miR-196a, miR-27a, miR-206)	Osteogenic gene regulatory networks	↑ Mineralization and osteogenic gene expression
Osteocytes	hUCB-MSC–Exos	Wnt/β-catenin (Wnt3a)	↑ ALP activity; enhanced osteocyte-mediated bone formation
Endothelial cells	MSC-Exos	HIF-1α–VEGF–ANG signaling	↑ Angiogenesis; coupling of angiogenesis and osteogenesis
Immune cells (T cells, macrophages)	miR-223 and immuno-modulatory cargos	Osteoimmune regulatory pathways	↓ Th1/Th17; ↑ Treg and M2 macrophages; reduced inflammatory bone loss

## Data Availability

No new data were created or analyzed in this study. Data sharing is not applicable to this article.
